# Case of the Dislocation of the Humerus

**Published:** 1870-05

**Authors:** T. A. Montgomery

**Affiliations:** Medical Student; Mankato, Minn.


Article V. — *Case of Dislocation of Humerus.* By T. A. Montgomery, Medical Student; Mankato, Minn., March 3, 1870.

Mr. D. H. Tyner, ex-sheriff of Blue Earth county; aged 50; healthy. On Saturday, February 12th, while carrying wood up a flight of stairs, he fell backwards; striking his weight on his right hand and arm, which he had thrown behind him, to break his fall. On February 16th, four days after the receipt of the injury, he made his appearance at the office of my preceptor, Dr. W. W.

Clark; stating, as his reason for not coming before, that he did not think the injury serious. He said he had experienced numbness of the hand and arm, from the first; with inability to raise it, or bring it forward across the chest. On examination, the injured shoulder appeared somewhat higher than that of the opposite side. There seemed to be an unnatural prominence of the acromion, with a hollow under it, at the back of the joint; also an undue fullness in front.

The diagnosis was partial dislocation of the humerus, upwards and forwards; the head of the bone lying against the coracoid process.

An attempt was immediately made to reduce it, but, owing to the rigidity of the muscles about the joint, arising from the length of time that had elapsed since the receipt of the injury, it proved ineffectual. He was then allowed about half an hour's rest, when, with the assistance of four strong men, the effort was again renewed. The patient was placed on a low couch and the shoulder fixed by a jacktowel, under the axilla; the ends being placed in the hands of assistants. Another towel was fastened to the arm by means of a clove hitch. In this way extension was made and continued. The surgeon grasping the arm, directed the assistants, while making the extension to raise the elbow toward the face, and at the same time, using the forearm as a lever, rotated it. By persevering in this manner for some time, the reduction was finally accomplished.

I have failed to find but two instances of this dislocation, either in the text books, or in any journal of medical or surgical science.

The patient is now out, attending to his regular business, and is able to use the joint quite freely, although the tendon of the biceps is, undoubtedly, out of the groove.

Moral.—Beware of procrastination.

